# Study Protocol – Diabetes and related conditions in urban Indigenous people in the Darwin, Australia region: aims, methods and participation in the DRUID Study

**DOI:** 10.1186/1471-2458-6-8

**Published:** 2006-01-17

**Authors:** Joan Cunningham, Kerin O'Dea, Terry Dunbar, Tarun Weeramanthri, Paul Zimmet, Jonathan Shaw

**Affiliations:** 1 Menzies School of Health Research and Institute of Advances Studies, Charles Darwin University, PO Box 41096, Casuarina, NT 0811, Australia; 2 Charles Darwin University, Darwin, NT 0909, Australia; 3 Northern Territory Department of Health and Community Services, PO Box 40596, Casuarina, NT 0811, Australia; 4 International Diabetes Institute, 250 Kooyong Road, Caulfield, VIC 3162, Australia

## Abstract

**Background:**

Diabetes mellitus is a serious and increasing health problem in Australia and is a designated national health priority. Diabetes and related conditions represent an even greater health burden among Indigenous Australians (Aborigines and Torres Strait Islanders), but there are critical gaps in knowledge relating to the incidence and prevalence, aetiology, and prevention of diabetes in this group, including a lack of information on the burden of disease among Indigenous people in urban areas. The DRUID Study (Diabetes and Related conditions in Urban Indigenous people in the Darwin region) was designed to address this knowledge gap.

**Methods/design:**

The study was conducted in a specified geographic area in and around Darwin, Australia. Eligible participants underwent a health examination, including collection of blood and urine samples, clinical and anthropometric measurements, and administration of questionnaires, with an additional assessment for people with diabetes. The study was designed to incorporate local Indigenous leadership, facilitate community engagement, and provide employment and training opportunities for local Indigenous people. A variety of recruitment methods were used. A total of 1,004 eligible people gave consent and provided at least one measurement. When compared with census data for the Indigenous population living in the study area, there was a marked under-representation of males, but no substantial differences in age, place of residence, Indigenous group, or household income. Early participants were more likely than later participants to have previously diagnosed diabetes.

**Discussion:**

Despite lower than anticipated recruitment, this is, to our knowledge, the largest study ever conducted on the health of Indigenous Australians living in urban areas, a group which comprises the majority of Australia's Indigenous population but about whose health and wellbeing relatively little is known. The study is well-placed to provide new information that can be used by policy makers and service providers to improve the delivery of services and programs that affect the health of Indigenous people. It also represents a valuable opportunity to establish an urban Indigenous cohort study, provided participants can be followed successfully over time.

## Background

Diabetes mellitus, predominantly type 2, is a serious and increasing health problem in Australia and has been designated a national health priority area [1]. AusDiab, the first Australian national diabetes and lifestyle study, showed a diabetes prevalence of 7.4% in adults aged 25 years and over in 1999–2000, one of the highest for a Western nation [2]. Diabetes and related conditions represent an even greater health burden among Indigenous Australians (Aboriginal and Torres Strait Islander people). A number of studies in Indigenous communities in rural and remote areas have shown that the prevalence of diabetes is higher and the age of onset lower than among Australians overall [3,4]. Because the risk of complications increases with the duration of diabetes, an earlier age of onset means that Indigenous people with diabetes are likely to have significantly worse health than non-Indigenous people with diabetes of the same age.

Indigenous adults aged 25–54 years were hospitalised for diabetes at about 15–18 times the rate for non-Indigenous adults in 2001–02; death rates from diabetes among those aged 35–54 years were 27–35 times higher for Indigenous than non-Indigenous people in 2000–02 [5].

According to an extensive review of diabetes in Aboriginal and Torres Strait Islander populations [4], there remain critical gaps in knowledge relating to the incidence and prevalence, aetiology, and prevention of diabetes in this group. Among the areas highlighted by the review were: the lack of information on the burden of disease among Indigenous people in urban areas; the inadequacy of data on the prevalence and incidence of diabetes-related complications; and the relative lack of evaluations of the effectiveness of lifestyle interventions in preventing diabetes and cardiovascular disease in Indigenous populations. The DRUID Study (Diabetes and Related conditions in Urban Indigenous people in the Darwin region) was designed to provide important new information in each of these areas, although the extent to which it was able to do so was variable.

## Methods/design

### Aims & objectives

The main aims of the DRUID Study were to:

• provide the first accurate assessment of the burden of diabetes, diabetic complications and associated chronic diseases in an urban Indigenous population, and to make comparisons with Australian-wide data;

• work with an urban Indigenous community to plan, implement and evaluate interventions to reduce the incidence of diabetes among those at high risk;

• facilitate contact with, and use of, appropriate services for people with diabetes and related disorders;

• increase awareness and understanding of diabetes among Indigenous people in the Darwin region;

• facilitate community engagement and provide education, employment and training opportunities for Indigenous people in an urban area;

• increase the capacity of Indigenous people to prevent diabetes; and

• increase the capacity of Indigenous people with diabetes and related disorders to manage their health in partnership with health service providers.

The study was intended to have three parts. The first part was baseline data collection, which combined recruitment and a health examination. This component, which is the subject of this paper, was expected to take approximately one year, but took approximately 50% longer, primarily due to slower than anticipated recruitment, as is discussed in detail below. The second component, a healthy lifestyle intervention among people with impaired glucose tolerance (IGT) and impaired fasting glucose (IFG), was expected to take place in years 2–5 of the study. This component was subsequently cancelled because the lower than expected number of total participants resulted in insufficient numbers of study participants with IFG or IGT. The third component – annual follow-up of participants to collect information on their vital status, incidence of selected major conditions and use of health services – is soon to commence.

In addition to undertaking formal data collection activities, it was intended that the study would increase diabetes awareness and provide opportunities for health education and health promotion, both through the provision of results and targeted health information to participating individuals, and through partnerships among researchers, service delivery organisations and advocacy bodies.

### Setting and location

The study was conducted in and around Darwin, a city of approximately 100,000 people located on the northern coast of Australia. At the time of planning the study, Darwin had a number of characteristics that were expected to greatly enhance the feasibility of the study and the identification, recruitment and follow up of participants, such as: perceived community support for, and potential involvement in, the study; strong and stable family networks; excellent identification of Indigenous people in the relevant administrative collections; a relatively high number of Indigenous people in a relatively small total population; existing partnerships between researchers and health practitioners through the Cooperative Research Centre for Aboriginal Health and other forums; and a pool of local Indigenous people who had research experience and/or could be trained to participate actively in the conduct of the study. In addition, the Darwin Indigenous population was similar to the total Australian Indigenous population on a range of socio-demographic and health risk factor variables, such as unemployment rate, post-school qualifications, household size, proportion of households in rented accommodation, smoking status, and proportion overweight/obese [6,7]. This was expected to greatly enhance the generalisability of the study.

### Funding

Major funding was provided by the National Health and Medical Research Council of Australia (project grant #236207). Supplementary funding was provided by the Vincent Fairfax Family Foundation, the Clive and Vera Ramaciotti Foundation (equipment grant # RA040/03), the Australian Government Department of Employment and Workplace Relations, and the AusDiab Partnership in type 2 diabetes. Several organisations provided in-kind support, as described below.

### Organisational structure

The Study was intended to be run as a partnership involving researchers, health service providers and Indigenous people and organisations. A number of groups were formed to support this partnership approach, including a management group, an Indigenous Steering Group (ISG), a technical reference group (TRG), and the project team. In order to facilitate communication, there was cross-representation across these groups.

The management group, comprising investigators and the project manager, had ultimate financial and scientific responsibility for the study overall. The ISG was intended to represent Indigenous groups in the local area and provide leadership and ensure practical benefits to the Indigenous community. Members were primarily Indigenous residents of the Darwin region who had a strong interest in health issues and who saw diabetes as an important issue for themselves, their families and their community. The TRG was intended to provide technical expertise and guidance and contribute to the rigor and efficiency of the study. Most of the work of this group was done in smaller groups on an ad hoc basis. Members included researchers and service providers chosen on the basis of their expertise and interest in the study. The project team was responsible for the day to day management and implementation of the study in keeping with broad directions set by the management group and the ISG.

A number of organisations were identified as project partners. These organisations indicated their support for the study and committed themselves to providing direct and/or in-kind support in various ways, including staff time, facilities, expertise and other support, as indicated in table 1.

Several other local organisations, such as community organisations, workplaces, and high schools, also made important contributions to the study, most commonly through assisting in recruitment and providing facilities for screening. Bayer HealthCare provided two urinalysis machines and accompanying consumables.

### Study principles

DRUID study partners committed to several core principles, including: developing and maintaining trust; Indigenous direction; employment, training and development opportunities for Indigenous people; high quality research methods; a transdisciplinary research focus; openness to working in new and appropriate ways; evaluation; and research transfer.

### Ethics

The study was approved by the joint Menzies School of Health Research – Northern Territory Department of Health and Community Services Human Research Ethics Committee. The project was considered and approved by both the Aboriginal sub-committee, which has absolute right of veto, and by the main committee.

### Target population

The study was aimed at adults aged 15 years and over who identified as Aboriginal and/or Torres Strait Islander, had lived within a geographically defined area around Darwin for at least 6 months, and who lived in a private dwelling (i.e., who did not live in an institution, such as a hospital, prison, boarding school or nursing home). The defined area was an administrative region known as the Yilli Rreung Aboriginal and Torres Strait Islander Commission (ATSIC) Region. This region of approximately 10,355 square kilometres is located in the northwest corner of the Northern Territory and includes the cities of Darwin and Palmerston, and a rural hinterland with several small towns and communities.

Based on data from the 2001 Census, the estimated resident Indigenous population (ERP) of the Yilli Rreung ATSIC Region as at 1 July 2004 (the approximate mid-point of the recruiting phase) included approximately 12,306 Indigenous people. This ERP figure takes into account adjustments for people whose Indigenous status was not stated, the estimated net census undercount, and births, deaths and migration between the time of the Census and the time of the estimate [8,9]. Of these estimated 12,306 Indigenous people, approximately 7,861 were aged 15 years or more. The number living in private dwellings is not known precisely, but it can be estimated using data from 2001 Census counts. Approximately 89.7% of those enumerated in the Yilli Rreung ATSIC Region were enumerated in private dwellings [10]. If this figure is applied to the Indigenous ERP, then approximately 7,051 people were potentially eligible based on age, Indigenous status and type of dwelling. It is not known what proportion of this group had lived within the Region for at least six months; the true number of eligible people is likely to be less than this figure.

### Participant recruitment

One of the main barriers to the conduct of population-based research in Australian Indigenous communities in urban areas is the lack of a sampling frame from which to select and contact potential participants. Indigenous people are not identified as such in the lists traditionally used in research, such as the electoral roll, telephone listings, etc. Although some existing lists do include Indigenous identification, these are usually incomplete and/or include people from outside the area of interest (e.g. Aboriginal Health Services client lists). In any case, current privacy laws do not generally allow this information to be provided to researchers. In discrete Indigenous communities (generally in remote areas), it is possible to draw a sample based on total population lists and then exclude the small proportion of non-Indigenous residents. In most urban areas, by contrast, Indigenous people make up a relatively small proportion of the population and they are widely dispersed across the city.

Given the absence of a list from which to select a sample of participants, and the apparent support for and interest in the study at the time it was first proposed, it was decided to attempt to recruit as many eligible people as possible. Three main methods of recruiting participants were identified during the initial planning of the study, including: 1) recruiting through the local Aboriginal Health Service; 2) using community and family networks starting with members of the study's ISG and working outward; and 3) contacting people through the Northern Territory Department of Health and Community Services (NTDHCS). A full-time recruitment officer was appointed to assist in developing and implementing these and other complementary strategies.

None of the three main recruitment methods was an unqualified success. The Aboriginal Health Service (AHS) was going through a prolonged period of instability, with management problems, financial difficulties [11] and high staff turnover. This made it difficult for the AHS to deliver its core business. In such circumstances, it was not surprising that engagement with the DRUID Study was not its highest priority. Following repeated attempts to ascertain how the DRUID Study could assist the AHS while at the same time recruiting study participants, a DRUID staff member who is a diabetes educator was eventually seconded to the AHS to provide part-time diabetes education at no cost while also recruiting participants for the study from among the general clinic population.

The ISG members were mixed in their ability to recruit participants through their community and family networks. Several members of the group did not themselves participate in the study, while others referred scores of participants. The members of the group, who were acting in a voluntary capacity, are leaders in their communities; as such, they face a large number of competing priorities for their limited time and energy. It is also possible that the long lead time between applying for and receiving funding led to a diminution of the momentum among this group. In any case, it was often difficult to ensure a quorum at ISG meetings once the study was up and running.

Contacting potentially eligible people through the NTDHCS was also limited in its success, in large part because of problems relating to out-of-date or incorrect addresses. The NTDHCS maintains a Client-Master Index which includes the name, Indigenous status and last known address of all people who have accessed public health or community services in the Northern Territory; this includes being born in an NT public hospital. (Most hospital services to Indigenous people in the NT are provided by public hospitals; there is one small private hospital in Darwin, but it has traditionally admitted relatively few Indigenous patients.) Although study staff could not legally access the Client-Master Index directly, it was agreed that the NTDHCS would send a letter and information packet on behalf of the DRUID Study to all people who appeared to be eligible for the study, based on Indigenous status, age and residential address. Of 8,061 letters sent out by NTDHCS, approximately 2,620 (33%) were returned to sender. Of these, the majority (68%) were reported as having moved. Others were returned to sender due to an unknown or insufficient address (24%), were unclaimed (7%), or the addressee was reported as deceased (2%). It is highly likely that other letters did not reach their intended destination and were destroyed rather than returned, but it is impossible to be certain of the magnitude. Several DRUID staff members who were eligible to participate in the study did not receive one of these letters.

A variety of other recruitment strategies was used throughout the study, with varying degrees of success. Some of these were aimed at increasing awareness of the study, while others were aimed at securing actual participation. A logo and slogan were designed, and a community launch was held during the first month of screening, with hundreds of key community members and organisations invited. Information packets were sent to all general medical practitioners and health centres in the area, explaining the study and encouraging them to refer all Indigenous patients. Booths were set up in major shopping centres, health fairs, sporting venues and at community events. Several newspaper ads were placed, especially at the beginning and end of the recruitment period. A television ad was made using a well-known local Indigenous musician. This was run as paid advertising on the main commercial station and as a community service announcement on both local commercial stations. A complementary radio ad campaign was run in conjunction with the television ads. Media releases were prepared to coincide with several events, resulting in stories with photos in several newspapers, as well as television news items and radio interviews. Information was also sent to the newsletters of sporting clubs and associations, employment groups and community organisations.

Translating general awareness of the study into either participation or a clear refusal to participate was especially challenging due to the lack of a list of names and contact details. Staff members entered potentially eligible people known to them into a database, but the number of entries fell far short of the estimated number eligible. Attempts were made to contact all those listed in the database by telephone or letter, but many could not be reached. Door-knocking was conducted in the two areas with the highest proportion of Indigenous residents, but this resulted in few completed appointments and was not continued.

Greater success was achieved through a series of visits to workplaces, high schools and discrete Indigenous communities, including town camps and small settlements. An estimated 40% or more of all participants were recruited through such visits. Recruitment and screening were conducted at approximately 20 workplaces (primarily NT and Australian Government agencies and community service organisations), five high schools, and nine discrete communities. The NT Public Service Commissioner agreed that eligible staff in NT Government departments could participate in the study during work time without loss of pay; other workplaces appeared to adopt a similar policy. Some supportive individuals took advantage of the study's offer to host family events as part of recruitment. Family members were invited to participate in screening at their relative's house, followed by a barbecue with food supplied by the study.

### Staffing and training

A total of 18 different individuals were employed on contracts over the course of the baseline phase of the study. Of these, 13 were Indigenous and 5 were non-Indigenous. Additional people were employed on a casual basis during visits to discrete communities; these community members acted as local facilitators. An Aboriginal Eye Health Worker from the local Aboriginal Health Service was seconded to the study during follow-up screening among participants with diabetes (approximately 1 day a month). Three post-graduate research students (one of whom is Indigenous) also participated in the study, with one substantially involved in data collection, and the other two participating in a more limited way.

All DRUID staff members who had access to data collected in the study were required to sign a confidentiality pledge. This pledge states that the person who signs it is aware of the sensitive and confidential nature of the information, and that he or she understands that inappropriate use of the information will be grounds for immediate disciplinary action, which may include termination of employment. This pledge was based on that used in the Strong Heart Study in the US [12].

A DRUID Study staffing and recruitment strategy was developed in conjunction with the ISG. In addition to traditional recruitment methods such as newspaper advertising, information on the positions available was sent for wide distribution to all ISG members as well as to a number of local community organisations and government departments. Secondments and flexible working arrangements were strongly encouraged, but these were not taken up. ISG members participated in drafting the position descriptions, and every selection panel included at least one member of the ISG.

Twelve staff were hired and trained together just prior to the start of data collection. Four staff had previously completed an Aboriginal Health Worker course, one was a nurse, and one had a certificate in laboratory skills. The others came from a variety of non-health backgrounds. With the exception of the project manager, none of the staff had a university degree, and few had completed high school. Only one staff member other than the project manager had previous research experience.

Training consisted of both on-the-job and formal training. This began with a one-week structured orientation/induction program including basic training in research methods and in diabetes understanding, followed by one week of specific role-based training. Staff were classified as having clinical, laboratory, interviewer or administrative responsibilities and were provided training relevant to these areas.

One of the activities included during the initial training program was the production of a video about the study. This video was used during the consent process and showed potential participants what would be involved, should they choose to take part. The successful production of the video required the actors (DRUID staff members) to learn to perform their study tasks to an adequate standard. This provided incentive to learn the material being presented to them during didactic instruction. During this same time, staff members also participated in discussions about suitable logos and slogans for the study and provided feedback on the data collection forms.

Over the course of the study, several staff members were supported to undertake additional training activities. For example, several staff completed a venipuncture certification course (including two who did not previously have clinical responsibilities), one enrolled in a post-graduate diabetes educator course, another in a Certificate 3 laboratory skills course, and another in undergraduate science units. Staff also attended a local conference on chronic disease as a group. Other informal training, mentoring and support were provided to all staff. Support for many of these additional training activities was provided by the Australian Government Department of Employment and Workplace Relations, through a Structured Training and Employment Project funded as part of the Government's Indigenous Employment Strategy.

The original staff members were hired on one-year contracts. This caused difficulty when recruitment was much slower than anticipated, because the contracts could not be shortened or terminated. With the exception of the initial project manager (who decided to pursue a PhD), staff who left the team prior to the expiry of their contracts were not replaced. These departures occurred for a variety of personal, health and performance related reasons.

### Consent

Potential participants were provided with written information about the study, invited to watch a video detailing what was involved, and given an opportunity to ask questions. Those who indicated that they wished to participate were then asked to complete and sign a consent form. A parent or guardian was asked to sign the form as well if the participant was under 18 years old.

Participants were asked to give separate consent for various elements of the study, including: questionnaires; fasting blood sample; 2-hour oral glucose tolerance test; urine sample; body measurements; blood pressure; and cardiovascular measurements. They were also asked to indicate the preferred method of contact (telephone or post) for their individual results, and whether they wanted a report sent to a primary health care provider (and if so, which one).

Participants were requested to select one of three options regarding the long-term storage of excess blood and urine samples: 1) destroy all remaining samples after completion of baseline tests; 2) store any remaining samples, but contact the participant to seek permission if a researcher wishes to use them in the future; 3) store any remaining samples, and use them without contacting the participant, provided their use is approved by the ISG and the relevant ethics committee(s).

Permission was also sought to obtain information about participants from other data sources, including: their health care provider(s); pathology services; the NT Department of Health and Community Services (for public hospital admissions); the Darwin Private Hospital; and the NT Registrar of Births, Deaths and Marriages and the National Death Index. This was necessary because of privacy legislation governing the secondary use of data from these sources.

The consent form indicated that study staff would contact participants in future years to update their contact details and to collect limited additional data on health and use of health services. Participants were asked to provide permission to contact their family and/or friends to help us find them in the future. This was intended to reduce suspicion and unwillingness to provide information by indicating the person's wishes to his or her family and friends.

We also asked participants whether they would be willing to have the study team contact them in the future: 1) to invite them to participate in a continuation of the current study beyond the initial five years; and 2) to discuss other related studies that have been approved by the study's ISG and by the relevant ethics committees.

Participants with diabetes who were invited to participate in additional tests for diabetes complications (described below) were asked to complete a second consent form. This form asked them to agree to participate in the various components of the complications assessment, including an eye examination, foot examination, blood pressure measurements, echocardiograph, and questionnaire. They were also asked to indicate how they wished to receive their results and whether they wanted a report sent to a health care provider (and if so, which one).

### Examination protocol

Following confirmation of contact details and eligibility, and after obtaining informed consent, a health examination was undertaken. This examination included: collection of blood and urine samples; clinical and anthropometric measurements; and the administration of questionnaires. The examination required a minimum of 2.5 hours for most participants, and was followed by a light lunch. After the examination was completed, participants were asked to provide contact details for any friends and relatives who might be interested in participating in the study.

People with diabetes were invited to return on a later day for additional tests, including examination of the eyes and feet, additional blood pressure measurements, an echocardiograph and additional questions relating to the management and complications of diabetes. This examination took approximately 30–45 minutes.

In order to determine the participant's fasting status and whether an oral glucose tolerance test was required, a few screening questions were asked regarding time since the person had last eaten, whether pregnant (females), and whether currently using insulin and/or tablets for diabetes. Oral glucose tolerance tests were not conducted on pregnant women or those currently using insulin and/or tablets for previously diagnosed diabetes. Those who had not fasted for at least 10 hours were asked to return on another day.

#### A) Blood and urine samples

For participants who reported fasting for at least 10 hours, a fasting blood sample was drawn, with up to 4 tubes (up to 13 ml) of blood collected. Immediately following this, participants were given a 75 ml glucose drink (Scot Scientific Pty Ltd, Welshpool, WA, Australia) unless they were currently taking tablets and/or insulin for previously diagnosed diabetes, were pregnant, or did not consent to this part of the study. A second blood sample (approximately 2 ml) was collected 2 hours later.

A urine sample was collected during the visit, at a time of the participant's choosing. Urine samples were sent away for analysis as described below. In addition, local dipstick testing of urine was introduced after the first few months of recruitment to establish whether a urinary tract infection was present at the time of the examination. Dipstick testing was performed using Multistix 10SG multiple reagent strips (Bayer Australia Ltd, Pymble, NSW, Australia) and a urine chemistry analyser (either a Clinitek 50, Bayer Corporation, Elkhart, IN, USA or a Clinitek Status, Bayer HealthCare Diagnostics Division, Sudbury, Suffolk, UK). Participants with urine that was dipstick-positive for leucocytes, nitrites and/or blood were asked for permission to send a midstream urine sample to a local pathology service for further testing, with results provided to the DRUID study. If positive, both the participant and his or her general medical practitioner were notified. Some participants declined to give permission for further urine testing; they were asked to see their primary health care provider for follow-up.

Blood and urine samples were labelled, processed, frozen, and shipped to the Clinical Trials Laboratory at Flinders Medical Centre (Bedford Park, South Australia) for analysis. The instruments and analytical methods used were as shown in table 2.

Blood samples were stored on ice until centrifuged. With the exception of HbA1c (for which whole blood was used), all blood and urine was spun by centrifuge at 3000 RPM for 15 minutes. Whenever possible, blood samples were spun within one hour of collection.

As part of a sub-study on polycystic ovary syndrome (PCOS), an additional blood sample was collected, generally as part of the fasting sample, for females aged 45 years and under. These samples were processed in the same way as the other blood samples (as described above), but they were sent to the Repromed Laboratories (University of Adelaide, South Australia). Details on assays and analytic methods are shown in table 3.

#### B) Clinical and anthropometric measurements

Clinical measurements included blood pressure and pulse rate and, for participants aged 25 years and over, an electrocardiogram. Anthropometric measures included height, weight, waist circumference and hip circumference. No anthropometric measurements were taken for participants in wheelchairs, and only height was measured for pregnant women.

Blood pressure was measured three times after the participant had been seated quietly for at least 5 minutes. A Welch Allyn Spot Vital Signs monitor (Welch Allyn Medical Products, Skaneateles Falls, USA) was used to measure systolic and diastolic pressure, with one minute between readings. The pulse rate from the second reading was also recorded. For every twentieth participant, an additional measurement of blood pressure was taken using the auscultatory (manual) technique. For these participants, manual and automatic measurements were done in alternating order.

An electrocardiogram (ECG) was performed on participants aged 25 years and over using a Philips PageWriter 10i handheld 12-lead electrocardiograph with automatic interpretation (Philips Medical Systems, Andover, MA, USA). ECG tracings were evaluated and coded in a single batch by a trained and certified Minnesota coder.

Some participants aged between 30 and 60 years were part of a special sub-study involving measurement of carotid intima media thickness using ultrasound, and of arterial stiffness using pulsewave analysis. These methods will be described elsewhere.

Height in centimetres was measured using a portable stadiometer (Model PE87, Mentone Educational Centre, Moorabbin, Victoria, Australia). Participants were shoeless and wore light clothing. They were instructed to stand facing forward with weight distributed evenly on both feet, with heels together and against the wall and arms hanging loosely by their sides. The participant's head was positioned so that the Frankfort Plane was horizontal. The participant was then instructed to keep his or her eyes focused on a point straight ahead, breathe in deeply, and stretch to his or her fullest height, with heels still on the ground. The measuring plate was then lowered onto the scalp until it rested firmly on the top of the head and a reading to the nearest 0.1 cm was obtained and recorded. Following the measurement of weight (as described below), a second height measurement was obtained and recorded, again to the nearest 0.1 cm. If the two height measurements differed by more than 0.5 cm, a third measurement was taken and recorded.

Weight in kilograms was measured using a Seca digital portable scale (Model 767, Seca Deutschland, Hamburg, Germany). Participants were asked to remove shoes, heavy garments, heavy jewellery, belts, loose change, keys, mobile phones, and other items from pockets. With the scales at zero, participants were asked to stand on the centre of the base with feet together, arms hanging loosely at the sides and the head facing forward. Weight was recorded to the nearest 0.1 kilogram.

Waist and hip circumference were measured in centimetres using a 2-metre non-stretch fibreglass tape. Following removal of outer clothing, tight-fitting garments, belts and heavy items from pockets, participants were asked to stand comfortably erect in a relaxed manner, breathing normally, with weight balanced evenly on both feet, feet about 25–30 cm apart, and arms folded across the chest. Waist and hip measurements were taken alternately, with a minimum of two measurements for each. All measurements were taken and recorded to the nearest 0.1 centimetre. If the first two measurements of either waist or hip circumference differed by more than 1.0 cm, then a third measurement was taken for waist or hip, as relevant. Waist measurements were taken directly on the participant's skin if possible, or over at most one layer of clothing. Hip measurements were taken over the participant's clothing, unless it was too tight or too baggy, in which case the participant was asked to remove it.

The waist was defined as the midway point between the iliac crest and the costal margin. These two landmarks were identified and marked using a felt tip pen, and the distance between them measured, with the midpoint marked. Once the tape was around the participant's body at the appropriate height and the tape was horizontal, the participant was asked to breathe out gently. The measurement was taken at the end of a normal expiration, with the tape pulled snug but not compressing the underlying soft tissue.

The hip circumference was defined as the widest circumference over the buttocks and below the iliac crest. The hip circumference was measured at several positions (starting with the widest part of the buttocks when viewed from the side) and the widest circumference recorded. Fatty aprons were not included in the measurement of the hips. The measurement was taken with the tape in a horizontal position, and the tape was pulled to allow it to maintain its position without causing indentation.

#### C) Questionnaires

Questionnaires covered a range of topics, as indicated in table 4. Questionnaires were originally interviewer-administered due to concerns about English literacy in the target population, but many participants indicated that they preferred to read the questions themselves. Forms were revised after the first three months to allow either self-administration or interviewer administration. No changes to content or question wording were made at that time. A coding system was already in use to record the identification of the administrator, using unique DRUID interviewer codes; a new code was assigned to indicate self-administration.

In response to concerns about slower-than-anticipated recruitment, modifications were made to the questionnaires after the study had been underway for approximately six months. For participants assigned an ID number less than or equal to 400, three questionnaires covered the following areas: 1) General medical history and risk factors; 2) Psychosocial factors; 3) Women's reproductive health (females only). After six months, the first two questionnaires were shortened and combined into one form. The content of the women's reproductive health questionnaire was unchanged, but administration was limited to women aged 45 years and under.

Approximately 40% of Questionnaire 1 (Version 1) and Questionnaire 2 (Version 1) were self-administered. This increased to 91% of the combined Version 2 Questionnaire. About 79% of women's reproductive health questionnaires were self-administered.

#### D) Additional tests for people with diabetes

Participants with diabetes, either previously known or newly diagnosed through the study, were invited back for an additional set of tests relating to diabetes complications. For the purposes of this study, a participant was considered to have diabetes if: a) he or she reported a previous physician diagnosis of diabetes and was currently taking tablets and/or insulin; or b) his or her blood test results indicated glucose levels consistent with diabetes (i.e. fasting glucose greater than or equal to 7.0 mmol/L and/or 2-hour glucose greater than or equal to 11.1 mmol/L).

Diabetes complications screening sessions were held approximately every other month. Some participants attended the first session following their initial health examination, others attended only after several months of broken appointments and numerous reminder calls, and some declined to participate in this part of the study.

The complications assessment consisted of a questionnaire, a clinical examination, and an eye examination. The questionnaire covered the following topics: age at diagnosis, diabetes care provider, history of amputations, history of foot ulcers, signs and symptoms in the legs and feet (including the Edinburgh Claudication Questionnaire [13] and the Neuropathy Symptom Score [14]), most recent foot exam, use of footwear, use of specified medications, and history of eye problems related to diabetes.

The clinical examination included inspection for foot ulcers, assessment of ankle reflexes and sensation in the great toes [14], pressure perception threshold at three sites on each foot [15], lying and standing blood pressure [16], and measurement of ankle brachial pressure index [17].

Assessment of ankle reflexes was performed using a standard reflex hammer. Reflexes were examined with the participant kneeling on a chair. If reflexes were absent then the assessment was repeated with reinforcement (the participant gripping the chair & gritting his or her teeth at the same time). Reflexes were classified as normal, present with reinforcement or absent.

Vibration perception (on the great toes) and temperature perception (on the dorsal surface of the metatarsal heads) were assessed using a standard 128 Hz tuning fork. Pin-prick perception was assessed on the dorsal surface of the great toes using a sterile neurological examination pin (Neurotips, Owen Mumford, Woodstock, Oxford, England). Participants were tested with their eyes closed.

Pressure perception threshold was measured using a 10 g monofilament at the great toe, first metatarsal head and fifth metatarsal head on the plantar surface of each foot. Participants were tested with their eyes closed. The order of sites was varied from participant to participant.

Lying and standing blood pressure were measured using a manual anaeroid sphygmomanometer (Welch Allyn Medical Products, Skaneateles Falls, USA). Systolic and diastolic pressures were measured at the brachial artery in the right arm while the participant was in a supine position, after lying down for at least 5 minutes. The measurements were repeated one minute after standing.

Ankle brachial pressure index was measured with the participant in a supine position. Systolic arm pressure was measured at the brachial artery using a Doppler probe (Dopplex High Sensitivity Pocket Doppler Model D900, Huntleigh Healthcare Pty Ltd, Hamilton Hill, WA, Australia) and an anaeroid sphygmomanometer (Welch Allyn Medical Products, Skaneateles Falls, USA). This was followed by Doppler measurement at the dorsalis pedis or posterior tibial artery of the foot.

The eye examination included measurement of visual acuity and digital retinal photography. Visual acuity was measured using a standard 6-metre wall-mounted Snellen eye chart (BOC Ophthalmic Instruments, Pty Ltd, Silverwater, NSW). Participants were tested as they presented, i.e. wearing their glasses or contact lenses if they wore them to the testing site. Pinhole testing was performed for those whose presenting visual acuity was worse than 6/6, but who were able to at least count fingers. Digital retinal photographs were taken by a trained Aboriginal Eye Health Worker using a Topcon non-mydriatic retinal camera (Model TRC-NW5, Topcon Corporation, Tokyo, Japan). Photographs were taken in two fields per eye (macula centred and nasal to disc). In some cases, eye drops were required to dilate pupils in order to obtain a good image; this was noted. Photographs were graded by a single ophthalmologist at the Centre for Eye Research Australia (Melbourne, Australia). If photos could not be obtained (usually because the participant declined or because the Eye Health Worker was not available), the participant was asked to provide consent for the study team to obtain recent results from the relevant eye care provider.

An echocardiogram was performed as part of a sub-study on selected participants aged between 25 and 64 years old. The methods will be described elsewhere. Measurement of urinary albumin-creatinine ratio (ACR) and glycated haemoglobin were performed during the initial stage of the study; most participants with diabetes also underwent an ECG, as they were 25 years or older. These tests and measurements were not repeated during the second visit.

### Provision of individual results

The provision of individual results was an important part of the study's relationship with the community. Some feedback was provided at the time of testing, and some participants, such as those with very high blood pressure or with chest pains plus an abnormal ECG, were referred immediately to the appropriate health care provider. DRUID clinical staff also maintained a list of primary care providers for participants who reported that they did not have a usual care provider. Before the first individual results were sent, all local medical practitioners identified by the Top End Division of General Practice were provided with information on what the reports would contain, as well as information on when, how and where to refer patients with abnormal results and how to use relevant Medicare item numbers. This information was designed with the assistance and input of local medical specialists.

A packet containing individual results was provided to each participant once his or her blood and urine results were received from the Clinical Trials Laboratory. Participants received their own values for height, weight, body mass index, waist circumference, self-reported smoking status, blood pressure, total cholesterol, triglycerides, HDL cholesterol, urine ACR, glomerular filtration rate (GFR), and fasting and two hour glucose values (and HbA1c for people with previously diagnosed diabetes), along with a description of what the test or measurement indicated, the range of normal values and a comment on their own scores. Participants also received health education materials tailored to their specific results. With the participant's consent, a report was also sent to the person's primary health care provider. Whenever possible, participants with abnormal results were first contacted by telephone or in person. The results packet was then mailed or hand-delivered, as appropriate. Some results were not able to be delivered because the participant had moved or could otherwise not be located.

People who participated in the assessment of diabetes complications were provided with information about their foot examination on the day of testing. They also received information about diabetes and its complications from staff, including an endocrinologist specialising in diabetes and a diabetes educator. Participants received a letter indicating their eye results once the grading of retinal photographs was completed. With the participant's consent, a report on the complications assessment was sent to the participant's primary care provider and/or diabetes care provider. This report included information on foot ulcers, amputations, pressure perception threshold test, ankle brachial pressure index, urine ACR and GFR (from the initial examination), visual acuity, and retinopathy grading.

### Data handling and statistical methods

Information about potential participants was entered into specially modified business contact tracing software (Goldmine Business Contact Manager Edition with GoldSync, Version 6.0.21021, FrontRange Solutions Inc., Colorado Springs, CO, USA). Although it was intended to use this software for all appointment booking, recruitment activities and participant contact, the relative lack of intermediate-level computing skills within the team meant that the potential of this system was not fully realised.

All data other than name and contact details were entered, using a combination of manual entry and optical scanning, into a custom-designed Oracle database (Oracle NET8, Oracle Corporation, Redwood City, CA, USA) with a Microsoft Access front end. Anthropometric and clinical measurements were entered manually from the relevant recording form, as were responses to all questions on the consent forms. All questionnaires were scanned using Remark Office OMR^® ^version 5.5 software (Principia Products, Paoli, PA, USA) and a high-speed duplex colour image scanner (Fujitsu fi-4120C, Fujitsu Australia Ltd, North Ryde NSW, Australia). Blood and urine results and retinal grading were provided by the relevant laboratory in spreadsheets and uploaded directly to the Oracle database. All data will be subjected to a range of assessments relating to completeness, accuracy, and plausibility. Once these checks have been completed, a final data set will be created.

All statistical analysis will be performed using the latest available release of Stata software (Stata Corporation, College Station, TX). Techniques will vary according to the specific analysis, but will primarily involve multivariate linear or logistic regression.

### Study participants

Between 12 September 2003 and 31 March 2005, a total of 1,033 Indigenous residents of the Yilli Rreung ATSIC Region completed a consent form. Twenty-four of these participants were ineligible due to length of residency or age and were excluded from further consideration: 16 indicated that they had lived in the area for less than 6 months, and a further 8 high school students had not yet turned 15 years old. Study staff also tested 7 Indigenous people who were not residents of Yilli Rreung and 28 non-Indigenous volunteers (12 as part of initial training and another 16 who were mostly family members of Indigenous participants or DRUID staff members). The tests of people known to be ineligible were done in certain circumstances to promote a positive image of the study and to increase awareness of diabetes.

Of the 1,009 eligible participants who gave consent, 1,004 provided at least one measurement. The numbers of eligible participants who provided selected information was as follows: consent – 1,009; fasting blood sample – 945; oral glucose tolerance test – 815; urine sample – 910; questionnaire 1 (version 1 or 2) – 920; questionnaire 2 (version 1) – 312; women's reproductive health questionnaire – 441 (women aged 45 years and under); blood pressure – 996; height – 969; weight – 965; waist circumference – 938; and hip circumference – 935. Of the 175 participants eligible for complications assessment, 138 were examined.

### Comparison of participants with other estimates of the target population

The 1,009 eligible people who completed a consent form represent approximately 14.3% of the estimated 7,051 people who were potentially eligible as at 1 July 2004 (the approximate midpoint of the recruitment period). Information on sex, age group, area of residence, Indigenous status and household income was compared for DRUID participants and for Yilli Rreung residents, based on data from the 2001 Census and from the NTDHCS Client-Master Index. This is presented in the following figures.

**Figure 1:**
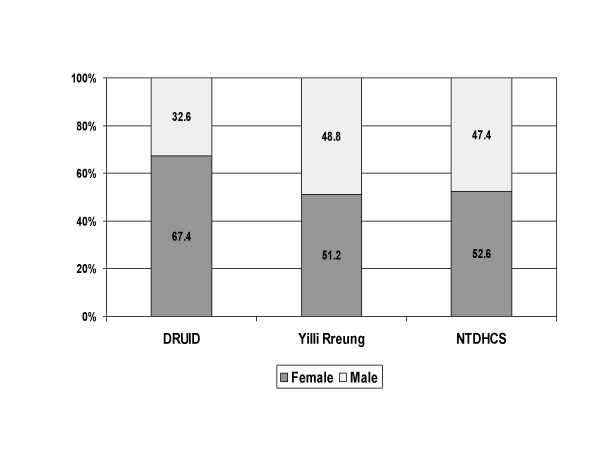
**Sex distribution: DRUID participants versus two comparison groups**. Comparison of DRUID participants with the 2004 Indigenous estimated resident population of the Yilli Rreung ATSIC Region [9] and with potentially eligible Indigenous clients from the Northern Territory Department of Health and Community Services' (NTDHCS) Client-Master Index.

As shown in figure 1, the proportion of females participating in the DRUID Study was substantially and significantly higher (p < 0.001) than the proportion in the Indigenous population of the Yilli Rreung region as estimated in 2004. It was also significantly higher (p < 0.001) than in the group identified as potentially eligible in the NTDHCS Client-Master Index.

**Figure 2:**
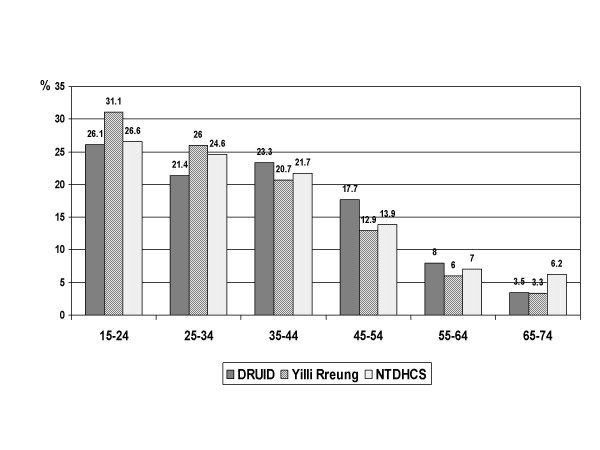
**Age distribution: DRUID participants versus two comparison groups**. Comparison of DRUID participants with the 2004 Indigenous estimated resident population of the Yilli Rreung ATSIC Region [9] and with potentially eligible Indigenous clients from the Northern Territory Department of Health and Community Services' (NTDHCS) Client-Master Index (CMI).

**Figure 3:**
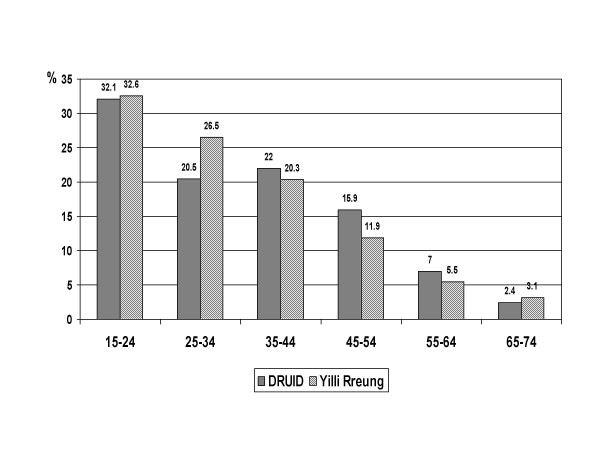
**Age distribution of males: DRUID participants versus Yilli Rreung residents**. Comparison of DRUID participants with the 2004 Indigenous estimated resident population of the Yilli Rreung ATSIC Region [9].

**Figure 4:**
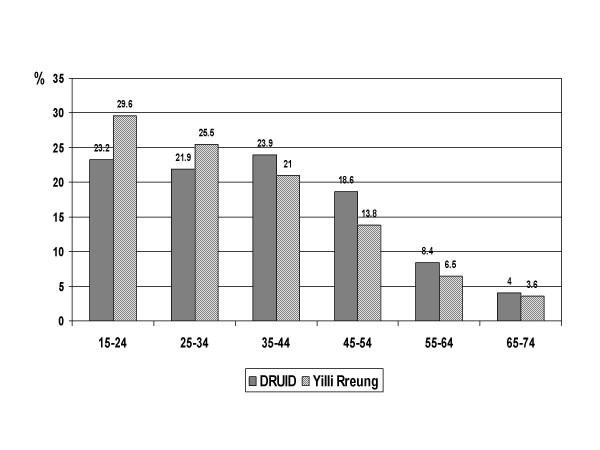
**Age distribution of females: DRUID participants versus Yilli Rreung residents**. Comparison of DRUID participants with the 2004 Indigenous estimated resident population of the Yilli Rreung ATSIC Region [9].

The age distribution was also significantly different in the DRUID Study than in the Yilli Rreung population in 2004 (p < 0.001) or the NTDHCS Client-Master Index (p < 0.001), with fewer than expected participants in the younger age groups (figure 2). Unlike the DRUID Study, the Yilli Rreung ERP and the NTDHCS group include people in non-private dwellings. Institutions such as boarding schools and prisons tend to include younger people, and this could explain at least some of the differences in age structure. The difference among males was relatively small except among those aged 25–34 years (figure 3), and was not significantly different (p = 0.065). Among females (figure 4), the difference (p < 0.001) was most marked among those aged 15–24 years.

**Figure 5:**
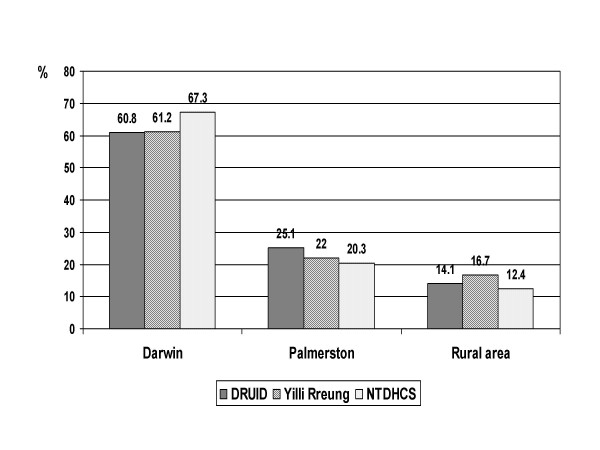
**Area of residence: DRUID participants versus two comparison groups**. Comparison of DRUID participants with 2001 Census counts for Indigenous residents of the Yilli Rreung ATSIC Region [10] and with potentially eligible Indigenous clients from the Northern Territory Department of Health and Community Services' (NTDHCS) Client-Master Index (CMI). DRUID and NTDHCS figures exclude people with missing or ineligible postal codes.

The distribution by place of residence was similar for DRUID Study participants and the Yilli Rreung Indigenous ERP (figure 5), although the small differences seen were statistically significant (p = 0.028) due to the large numbers in the population. The distribution of the NTDHCS Client-Master Index was somewhat different from the other two groups. This may reflect the fact that the Yilli Rreung ERP is based on place of residence, while the NTDHCS group is categorised by mailing address. The DRUID Study group reflects a combination of residential and mailing addresses.

**Figure 6:**
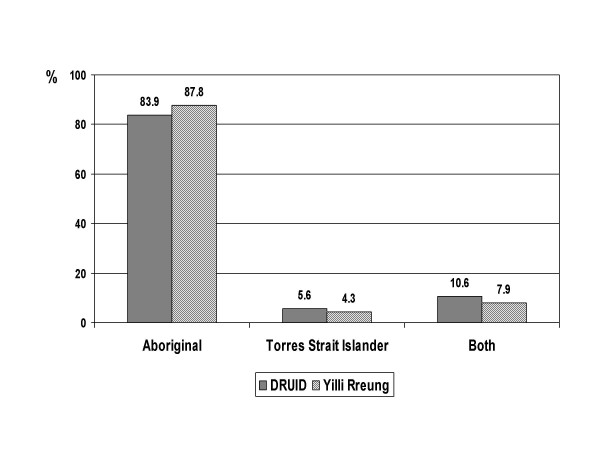
**Indigenous group: DRUID participants versus Yilli Rreung residents**. Comparison of DRUID participants with 2001 Census counts for Indigenous residents of the Yilli Rreung ATSIC Region [10].

The proportion of DRUID participants reporting sole Aboriginal identification was slightly lower than in the Yilli Rreung ERP, while the proportion reporting mixed (Aboriginal and Torres Strait Islander) origin was higher (figure 6). Although the differences were small, they were statistically significant (p = 0.001).

**Figure 7:**
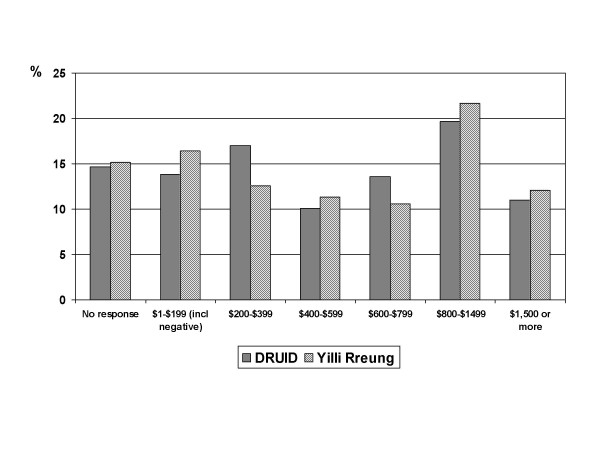
**Comparison of DRUID participants and Indigenous Yilli Rreung residents counted at the 2001 Census: weekly household income**. Comparison of DRUID participants with 2001 Census counts for Indigenous residents of the Yilli Rreung ATSIC Region [10]. Figures are in Australian dollars.

The distribution of weekly household income was also significantly different (p = 0.002), but not in any systematic way (figure 7). Some of the difference may reflect changes between the income levels reported on the 2001 Census (Yilli Rreung population) and those reported at the time of the DRUID Study in 2003–2005. The high proportion of people who did not provide a response makes comparisons somewhat difficult.

**Figure 8:**
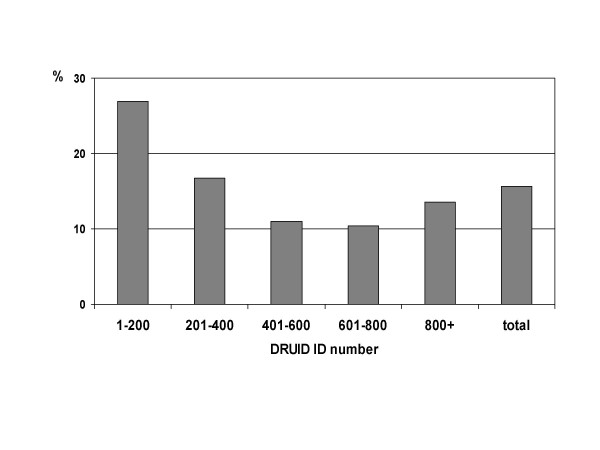
Proportion of DRUID participants reporting previously diagnosed diabetes, by ID number.

It is not possible to know whether the health status of DRUID Study participants was different from that of non-participants. There is some evidence to suggest that the health status of participants varied over the course of the study, with early participants significantly more likely (p = 0.001) to report previously diagnosed diabetes (figure 8). This is likely to reflect a widely held belief early in the recruitment period that the study was intended for people with diabetes, rather than for the population as a whole. Subsequent publicity about the study was designed to emphasise the importance of broad participation, and there was no significant difference by ID number in the proportion with previously diagnosed diabetes among those with an ID number greater than 200 (p = 0.297).

**Figure 9:**
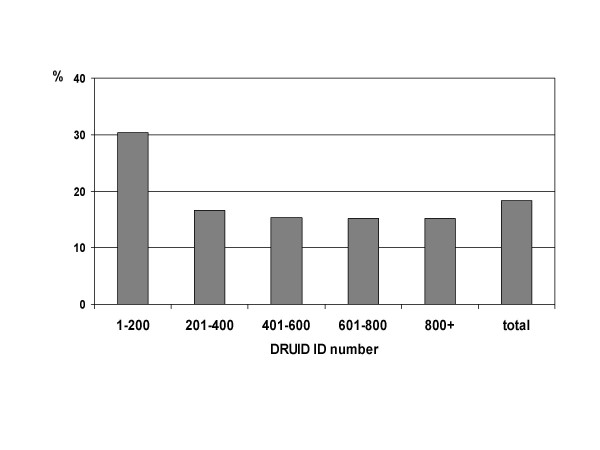
**Proportion of DRUID participants with hypertension, by ID number**. Hypertension includes measured blood pressure greater than or equal to 140/90 mm Hg and/or reported as currently taking antihypertensive medications.

**Figure 10:**
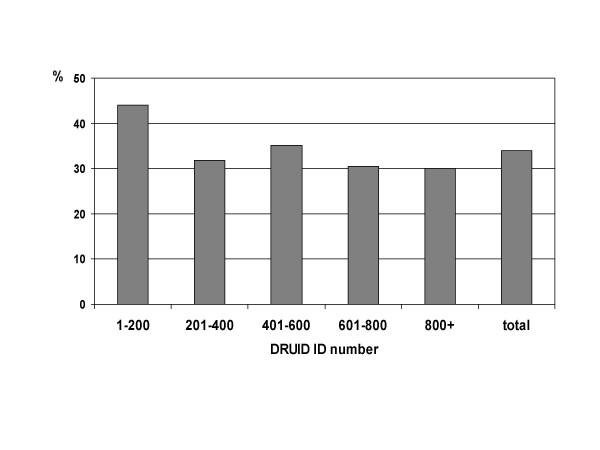
Proportion of DRUID participants with body mass index of 30 or more, by ID number.

As shown in figures 9 and 10, early participants were also significantly more likely to have hypertension (either measured blood pressure greater than or equal to 140/90 mm Hg, or currently taking antihypertensive medications; p < 0.001) and to have a body mass index classified as obese (p = 0.001). After excluding the first group of 200 ID numbers, there was no difference in the proportion with hypertension according to ID number (p = 0.982). There remained a significant difference in the proportion categorised as obese (p = 0.015), but in absolute terms, this difference was small.

**Figure 11:**
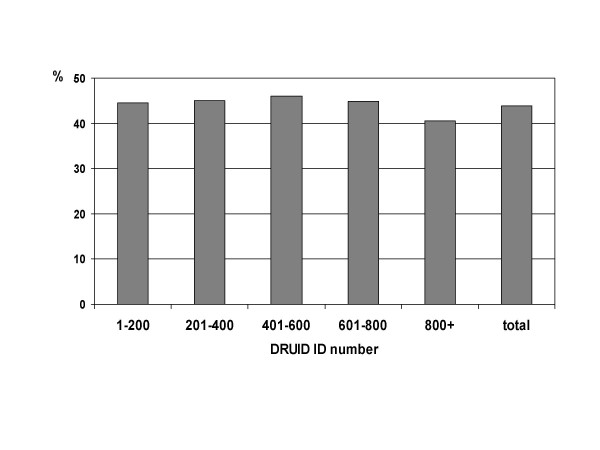
Proportion of DRUID participants reporting current smoking, by ID number.

The proportion of participants who reported they were current smokers was high as expected, and did not vary significantly over the course of the study (figure 11; p = 0.700).

**Figure 12:**
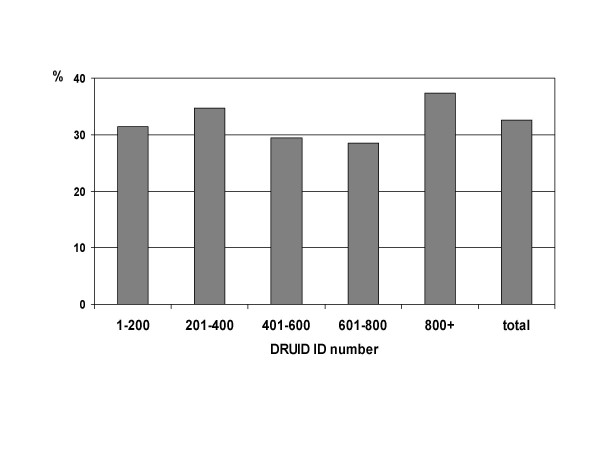
Proportion of DRUID participants who were male, by ID number.

**Figure 13:**
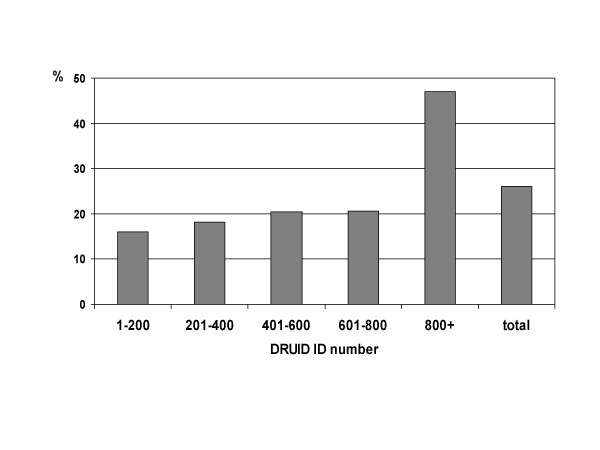
Proportion of DRUID participants in the age group 15–24 years, by ID number.

The proportion of males was not significantly different (p = 0.246) for earlier and later participants (figure 12), but there was a marked increase in the proportion of people aged 15–24 years old among later participants (figure 13; p < 0.001). This is due to the inclusion of several high schools during the last few months of recruitment.

## Discussion

Population health research involving Indigenous Australians has long been a challenging endeavour [18-20], and the DRUID Study is no exception. Despite apparent community support, interest and involvement in the study, the level of recruitment was well below that anticipated. As a result, we were unable to meet all of the initial aims of the study.

Our success in meeting our aim of providing the first accurate assessment of the burden of diabetes, diabetic complications and associated chronic diseases in an urban Indigenous population depends on how well the study participants represent the population of interest. Participants were not substantially different from the population of interest with respect to demographic features other than sex. Although there were statistically significant differences between study participants and the Yilli Rreung Indigenous estimated resident population in the distributions by age, sex, area of residence, Indigenous group and household income, these differences were small in absolute terms, with the notable exception of sex. In addition, the Yilli Rreung ERP includes people living in non-private dwellings, who were not eligible to participate in the study. This inclusion may explain some of the differences in the distributions between the two groups. In any case, it highlights the importance of using caution when interpreting such comparisons. The Australian Bureau of Statistics (ABS) does not produce a separate ERP figure for the Indigenous population in private dwellings, so the only other option using ABS data is to use census counts of people in private dwellings as the basis of comparison. However, unlike the ERP figures, these counts do not take into account people who were not included in the Census or who did not respond to the question on Indigenous status, and the figures are only available for years in which a census was conducted. As with the ERP, these factors could have an impact on the distributions of interest. Comparisons using the NTDHCS Client-Master Index must also be made with caution, given the high rate of letters returned to sender, and the fact that several eligible staff members did not receive a letter.

The representativeness of the health status of participants is unknown. Early participants were clearly more likely than later participants to have a history of chronic health problems, such as a previous diagnosis of diabetes and hypertension. However, after the first 200 participants, the rates generally stabilised, which may indicate that participation was independent of health status during most of the study. In addition, the proportion of participants who reported they were current smokers was stable throughout the study, which does not suggest any difference in health risk factor profiles, at least for one important health behaviour.

Although the disease prevalences observed in the study can not be interpreted as true population prevalences, they may still provide useful – albeit approximate and possibly somewhat biased – estimates. In any case, few alternative estimates are currently available for any urban Indigenous populations in Australia, and none exist for the Darwin region. Perhaps more importantly, the extensive data collected in the study will also be used to examine relationships among variables of interest. These data will provide information that is not currently available, and that can be used by policy makers and services providers to improve the services and programs that affect the health of Indigenous people.

The second aim of the study – to work with an urban Indigenous community to plan, implement and evaluate interventions to reduce the incidence of diabetes among those at high risk – was not able to be met due to inadequate numbers of participants with impaired fasting glucose and impaired glucose tolerance. This was a function of smaller than expected numbers of participants overall, rather than a lower than expected prevalence of IFG and IGT. In any case, the proposed intervention would have had inadequate power to detect important differences. It was therefore cancelled, with the relevant funds returned to the National Health and Medical Research Council.

We had greater success in relation to our other aims, although this is not always easy to demonstrate. The provision of individual results and advice and education materials to participants as well as interaction with staff members is likely to have increased awareness and understanding of diabetes among participants, although this has not been formally assessed. We were clearly able to provide employment and training opportunities for Indigenous people, with 13 Indigenous staff members over the course of the study, but facilitating community engagement proved an ongoing challenge. By engaging health service providers and referring participants with specific health problems to relevant services, we facilitated contact with, and use of, appropriate services for people with diabetes and related conditions. It is difficult at this point to determine the extent to which the study has contributed to achieving our longer term aims of increasing the capacity of Indigenous people to prevent diabetes and increasing the capacity of Indigenous people with diabetes and related disorders to manage their health in partnership with health service providers. Although there have been reports of some participants having adopted healthier lifestyles, and of people with diabetes becoming more involved in their health care following participation in the study, it is not clear how widespread this has been, nor whether any such changes have been maintained.

Despite the difficulties encountered in undertaking the DRUID Study, and our inability to achieve all of our original aims, the study was certainly not without its successes. We recruited over 1,000 participants, of whom approximately 94% provided a fasting blood sample. To our knowledge, this is the largest study ever conducted on the health of urban Indigenous Australians. As such it provides a valuable opportunity to establish a cohort study, providing enough participants can be contacted and followed over time. This will be assessed in the next phase of the study, which involves annual follow-up of participants to collect information on their vital status, incidence of selected major conditions and use of health services. If most participants can be traced and contacted, we will continue to follow the cohort over time, with a view to repeat examinations in the future. This would allow estimates of the incidence of diabetes and related conditions to be made, as well as enable a better understanding of changes over time. It would also see the original vision of the study achieved and would add exponentially to its value.

## Abbreviations

ABS Australian Bureau of Statistics

ACR albumin-creatinine ratio

AHS Aboriginal Health Service

ATSIC Aboriginal and Torres Strait Islander Commission

DRUID Diabetes and Related disorders in Urban Indigenous people in the Darwin region

ECG electrocardiogram

ERP estimated resident population

GFR glomerular filtration rate

HbA1c glycosylated hemoglobin

HDL high density lipoprotein

ID identification

IFG impaired fasting glucose

IGT impaired glucose tolerance

ISG Indigenous Steering Group

NT Northern Territory

NTDHCS Northern Territory Department of Health and Community Services

PCOS polycystic ovary syndrome

RPM revolutions per minute

TRG Technical Reference Group

## Competing interests

The author(s) declare that they have no competing interests.

## Authors' contributions

JC participated in the design of the study protocol, coordinated data collection and data management, analysed the data and drafted the manuscript. KO and PZ conceived of the study and participated in its design. TD chaired the study's Indigenous Steering Group, participated in the design of the study and facilitated Indigenous community input throughout the study. TW participated in the design of the study and provided clinical expertise during the design and data collection phases. JS participated in the study design and was responsible for the development of the diabetes complications assessment component. All authors were involved in revising the manuscript for important intellectual content and read and approved the final manuscript.

## Pre-publication history

The pre-publication history for this paper can be accessed here:



## Figures and Tables

**Table 1:** Contributions of DRUID Study Partner Organisations

Organisation	Contribution
Menzies School of Health Research	Two investigators, several ISG members, several TRG members, employment of the project team, three post-graduate research students, development of study protocols, provision of testing and office facilities, hosting of group meetings, garage and storage facilities, freezer space, general study infrastructure
Northern Territory Department of Health and Community Services	Two investigators, several ISG members, several TRG members, testing facilities at several locations, major mail-out to potential participants, input to study design, on-premises recruitment
Danila Dilba Health Service	Several ISG members, TRG members, Eye Health Worker for complications assessment, hosting of majority of ISG meetings, provision of occasional testing facilities, some recruitment
International Diabetes Institute	Two investigators, TRG members, provision of training for complications assessment, input to study design, advice on several aspects of the study including data management and laboratory setup, use of a proprietary software package, links with the AusDiab Partnership in Type 2 Diabetes and other national organisations
Cooperative Research Centre for Aboriginal Health	Two investigators, ISG members, assistance with development of the communications strategy, assistance with education and training issues
Yilli Rreung ATSIC Regional Council	ISG member (sitting fees were provided by the Council to enable attendance as part of official Council duties)
Diabetes Australia NT	TRG members, diabetes awareness training as part of staff orientation, educational resources, health education for participants at some complications screenings
Top End Division of General Practice	TRG members, facilitation of communication with general medical practitioners
National Heart Foundation (NT)	TRG member

**Table 2:** Analysis of blood and urine samples*

**Test**	**Anticoagulant**	**Instrument**	**Method principle**
Glucose – fasting, 2 hour	Fluoride/EDTA	Hitachi 917	Enzymatic
Insulin – fasting, 2 hour	Fluoride/EDTA	Abbott Axsym	Immunoassay
Homocysteine	Fluoride/EDTA	Agilent HPLC	Fluorometric
Cholesterol	None (serum)	Hitachi 917	Enzymatic
Triglyceride	None (serum)	Hitachi 917	Enzymatic
HDL-cholesterol	None (serum)	Hitachi 917	Enzymatic
LDL-cholesterol	N/A	Calculated	Calculated
Creatinine	None (serum)	Hitachi 917	Kinetic Jaffe
Hs-CRP	None (serum)	Hitachi 917	Immunoturbidimetry
HbA1c	EDTA	Agilent HPLC	Cation exchange
Fibrinogen	Sodium citrate	IL Futura plus	Clotting time
			
Urine albumin	None (urine)	Beckman Array 360	Immunonephelometry
Urine creatinine	None (urine)	Hitachi 917	Kinetic Jaffe
Urine ACR	N/A	Calculated	Calculated

* Note: Analysis of fatty acids and carotenoids using frozen samples is also planned. This is expected to occur in 2006. Methods will be described elsewhere.

**Table 3:** Analysis of blood samples for PCOS sub-study

**Test**	**Preservative**	**Instrument**	**Method principle**
Testosterone	Lithium heparin	n/a	RIA (radioimmunoassay)
Sex-hormone binding globulin SHBG	Lithium heparin	n/a	IRMA (immunoradiometric assay)
Free androgen index (FAI)	N/A	Calculated	Calculated
Free testosterone	N/A	Calculated	Derived from mass equations
17-hydroxyprogesterone (17-OHP)*	Lithium heparin	n/a	RIA (radioimmunoassay)
Prolactin*	Lithium heparin	Centaur	ICLMA (immunochemiluminometric assay)
Thyroid stimulating hormone (TSH)*	Lithium heparin	Centaur	ICLMA (immunochemiluminometric assay)

* These additional tests were performed for women with oligomenorrhoea plus at least one of the following: a positive questionnaire response to a question on problems with acne; a positive questionnaire response to a question on problems with hair; or FAI of 5.5 nmol/L or more. Women were categorised as having oligomenorrhoea if they reported irregular menses or reported that their menstrual cycles were either less than 24 days long or 35 or more days long. (Women who were post-menopausal or had undergone a hysterectomy were excluded.) All women in this group are currently being followed up as part of the PCOS sub-study.

**Table 4:** Topics included in DRUID Study questionnaires

**Topic**	Questionnaire Version*	
	**1**	**2**

Age and date of birth	Yes	Yes
Sex	Yes	Yes
Indigenous group	Yes	Yes
Time fasting	Yes	Yes
Whether pregnant	Yes	Yes
Whether previously tested for diabetes	Yes	Yes
Self-reported diabetes status	Yes	Yes
Current diabetes treatment (if applicable)	Yes	Yes
Usual health care provider	Yes	Yes
Medical history	Yes	Yes, but reduced
Diabetes care	Yes	No (but a few questions moved to diabetes complications form)
Dental health	Yes	No
Health insurance	Yes	Yes, but reduced
Alcohol use	Yes	Yes, but reduced and some questions changed
Smoking	Yes	Yes, but reduced and some questions changed
Physical activity	Yes	Yes, but reduced and some questions changed
Weight and nutrition	Yes	Yes
Subjective social status [21]	Yes	No
Household characteristics	Yes	Yes, but reduced
Education	Yes	Yes, but reduced
Employment	Yes	Yes, but reduced
Income	Yes	Yes, but reduced
Culture and identity	Yes	Yes
General health and wellbeing (SF-12) [22]	Yes	First question only
Stress	Yes	No
Discrimination	Yes	No
Depression (CES-D) [23]	Yes	No
Control	Yes	No
Social support [24]	Yes	No
Optimism/pessimism (LOT-R) [25]	Yes	No
For women only:		
Menstrual history	Yes	N/A§
Contraceptive use	Yes	N/A§
Pregnancy	Yes	N/A§
Infertility	Yes	N/A§
Hysterectomy	Yes	N/A§
Menopause	Yes	N/A§
Hormone replacement therapy	Yes	N/A§

* Version 1 was administered to participants with an ID less than or equal to 400. Version 2 was administered to subsequent participants.

§ The women's reproductive health questionnaire remained unchanged (i.e. Version 1), but administration was limited to women aged 45 years and under.
